# Antihypertensive properties of tilapia (*Oreochromis spp*.) frame and skin enzymatic protein hydrolysates

**DOI:** 10.1080/16546628.2017.1391666

**Published:** 2017-10-24

**Authors:** Hsin-Chieh Lin, Adeola M. Alashi, Rotimi E. Aluko, Bonnie Sun Pan, Yu-Wei Chang

**Affiliations:** ^a^ Department of Food Science, National Taiwan Ocean University, Keelung, Taiwan;; ^b^ Department of Human Nutritional Sciences, University of Manitoba, Winnipeg, MB, Canada

**Keywords:** Tilapia, frame protein hydrolysate, skin protein hydrolysate, angiotensin I-converting enzyme, renin, spontaneously hypertensive rats

## Abstract

Proteins from tilapia frame and skin can potentially be precursors of antihypertensive peptides according to the result of BIOPEP analyses. The aim was to generate peptides with inhibitory effects against angiotensin-converting enzyme (ACE) and renin from tilapia frame and skin protein isolates (FPI and SPI). The most active hydrolysate was then tested for blood pressure-lowering ability in spontaneously hypertensive rats (SHRs). Tilapia frame and skin protein hydrolysates (FPHs and SPHs) were respectively produced from FPI and SPI hydrolysis using pepsin, papain, or bromelain. The ACE-inhibitory activities of tilapia protein hydrolysates with varying degree of hydrolysis (DH) were evaluated. In order to enhance the activity, the hydrolysate was fractionated into four fractions (<1 kDa, 1–3 kDa, 3–5 kDa, and 5–10 kDa) and the one with the greatest ability to inhibit *in vitro* ACE and renin activities was subjected to oral administration (100 mg/kg body weight) to SHRs. Systolic and diastolic blood pressure (SBP and DBP), mean arterial pressure (MAP), and heart rates (HR) were subsequently measured within 24 h. The pepsin-hydrolyzed FPH (FPHPe) with the highest DH (23%) possessed the strongest ACE-inhibitory activity (IC_50_: 0.57 mg/mL). Its <1 kDa ultrafiltration fraction (FPHPe1) suppressed both ACE (IC_50_: 0.41 mg/mL) and renin activities more effectively than larger peptides. In addition, FPHPe1 significantly (*p* < 0.05) reduced SBP (maximum −33 mmHg), DBP (maximum −24 mmHg), MAP (maximum −28 mmHg), and HR (maximum −58 beats) in SHRs. FPHPe1 showed both *in vitro* and *in vivo* antihypertensive effects, which suggest tilapia processing coproducts may be valuable protein raw materials for producing antihypertensive peptides.

## Introduction

Tilapia is one of the most important and widely cultured food fish worldwide. Global aquaculture production of tilapia increased from 2 million tons in 2005 to 5.3 million tons in 2014 with an annual production growth around 10% [1]. During tilapia filleting, large quantities of by-products including tilapia head, frame, and skin, which may contain 16–80% protein content are underutilized [2,3]. As value-added usage of fish by-products has drawn attention, recovery or alteration of protein structure by enzyme technology has become a feasible choice [4].

Studies have revealed that hydrolysates or peptides derived from tilapia protein possess physiological functions. Enzymatic hydrolysates of tilapia muscle containing nearly 40% hydrophobic amino acids [5] showed antioxidant [6] and antihypertensive properties [7]. The preventive effect of tilapia hydrolysate against oxidative damage in HepG2 cells and the protective ability against DNA damage have also been reported [8]. In addition to the antioxidant and angiotensin converting enzyme (ACE)-inhibitory activities of tilapia frame [9] and skin gelatin [10] hydrolysates, tilapia protein hydrolysates also exhibited antibacterial activities [11]. Peptides from tilapia by-products displayed various biological activities as well. A calcium-binding peptide isolated from tilapia scale protein hydrolysate was discovered to prevent calcium deficiency with improved calcium bioavailability in rats [12].

The conventional approach of screening of bioactive peptides from different substrates is an inefficient process because it involves using empirical methods to select appropriate proteases and requires experimental evaluation of each one for *in vitro* activities. Due to advances in bioinformatics, this laborious process can be simplified by using in silico tools such as the BIOPEP database and computation program [13]. Such in silico methods have been widely applied for analyzing potential activities of food proteins after enzymatic digestion. For example, the ACE-inhibitory activities of protein hydrolysates from chickpea [14] and crude barley [15] were reported using this in silico approach. A similar approach was used to show that whey protein hydrolysate possessed both ACE-inhibitory and antioxidative effects [16]. Prolyl endopeptidase (PEP) inhibitory peptides were found to be present in bovine and porcine meat [17]. In our previous research work, some muscle proteins including myosin heavy chain (1523 amino acids) and alpha-actin (353 amino acids) were identified as the major proteins in tilapia (*Oreochromis spp*.) frame protein isolate (FPI), whereas skin protein isolate (SPI) was mostly composed of two types of collagen alpha chains (1450 and 1355 amino acids) [2]. Preliminary screening of encrypted bioactive peptides, identified based on the primary sequence of tilapia proteins, indicated that most of the peptides were ACE inhibitors. In addition, PEP-inhibitory peptides, which are related to antiamnesic activity were shown to be mainly present in SPI instead of FPI.

Renin and ACE are the two pivotal enzymes that regulate the renin-angiotensin system (RAS), which plays a crucial role in the pathogenesis of hypertension [18]. Angiotensinogen released from liver is cleaved by renin and generates angiotensin I, which is subsequently converted to angiotensin II by the action of ACE. Angiotensin II is a potent vasoconstrictor, which results in blood pressure increase. Thus, multifunctional peptides with simultaneous inhibition of renin and ACE activities provide a more efficient RAS regulation compared to specific single enzyme inhibitors [19].

Although the application of bioactive peptide database is efficient in choosing appropriate proteases, overlooking the interference factors associated with *in vitro* digestion could lead to discordance between in silico and *in vitro* methods [20]. In silico methods are used initially to assess the effectiveness of the enzyme-produced bioactive peptides. Further *in vitro* or *in vivo* studies are also required. After identification of the proteins from tilapia processing co-product using proteomic techniques, analysis of bioactive peptides based on the protein sequences was executed using BIOPEP. The in silico data revealed numerous ACE-inhibitory peptides within the primary structure of tilapia proteins. In this study, three commercial enzymes pepsin, papain, and bromelain were used to hydrolyze the proteins from tilapia processing co-products. Therefore, the objective of this work was to determine the *in vitro* inhibition of ACE and renin activities by the enzymatic hydrolysates of tilapia frame and skin proteins. The most active hydrolysate was then tested for blood pressure-reducing activity through oral administration to spontaneously hypertensive rats (SHR).

## Materials and methods

### Materials

Tilapia frame and skin were acquired from a local sea food processing plant (Fortune Life Company, Kaohsiung, Taiwan). Pepsin (from porcine gastric mucosa), papain (from *Carica papaya*) and bromelain (from pineapple stem) were purchased from Sigma-Aldrich (St. Louis, MO, USA). ACE (from rabbit lung) and its substrate, N-(3-[2-furyl]acryloyl)-phenylalanylglycylglycine (FAPGG) were also obtained from Sigma-Aldrich. Human recombinant Renin Inhibitor Screening Assay Kit was purchased from Cayman Chemicals (Ann Arbor, MI, USA). Other chemicals and reagents used were of analytical grade.

### Production of tilapia protein isolate

Preparation of tilapia frame protein isolate (FPI) and skin protein isolate (SPI) were based on the method of [7] and [21], respectively, with some modifications. Briefly, the tilapia frame was cut into pieces, homogenized with deionized water (1:9 w/v), the homogenate adjusted to pH 12 and then stirred at 4°C for 30 min. After filtration through a 10 µm mesh metal filter, the homogenate was centrifuged (8000 *g*, 4°C) for 10 min, the supernatant filtered through cheesecloth and adjusted to pH 5.5 to precipitate the proteins. The precipitate collected after another centrifugation round was lyophilized and labeled as frame protein isolate (FPI). Sliced tilapia skin was soaked in 95% ethanol (skin:ethanol, 1:9 w/v) for 48 h with a change in solvent after 24 h in order to remove lipid materials The defatted skin was then soaked for 6 h in 0.1 M NaOH (skin:NaOH, 1:9 w/v) followed by homogenization with deionized water (skin:NaOH, 1:9 w/v) and adjusted to pH 2. The homogenate was stirred at 4°C for 48 h and then centrifuged (8000 *g*, 4°C) for 20 min. The resultant supernatant was adjusted to pH 7, centrifuged and the precipitate lyophilized as skin protein isolate (SPI). Both FPI and SPI powder were each stored at −20°C until use.

### BIOPEP analysis of bioactive peptides from tilapia proteins

The assessment of potential antihypertensive and antiamnesic peptides from tilapia proteins was carried out using BIOPEP [22] analysis. Sequences of identified tilapia proteins were obtained from NCBI database as described previously [2]. Proteins were subjected to in silico papain or bromelain proteolysis to generate peptides with specific activities, which were then searched for and the numbers of ACE-inhibitory peptides calculated. In addition, pepsin, trypsin, and chymotrypsin A or C were applied simultaneously to cleave tilapia protein sequences in order to mimic protein digestion in the gastrointestinal tract.

### Enzymatic hydrolysis

Hydrolysis of FPI and SPI was conducted based on the method of Shamloo et al. [23]. FPI or SPI were homogenized with deionized water (protein isolate/water, 1:9 w/v) and adjusted to the optimal pH and temperature for each enzyme (pepsin: 37°C, pH 2; bromelain: 50°C, pH 7; papain: 55°C, pH 7). The hydrolysis was started by adding 1% (enzyme/substrate, w/w) protease. After 4-h hydrolysis of FPI or SPI using a single enzyme (pepsin, papain or bromelain), the digest was placed in a 95°C water bath for 15 min to inactivate the enzyme and then cooled to room temperature. The cooled digest was then centrifuged (10,000 *g*, 4°C) for 20 min and the supernatant was lyophilized and stored at −20°C as the frame or skin protein hydrolysate. Frame protein hydrolysates (FPHs) generated by pepsin, papain, or bromelain were designated as FPHPe, FPHPa, and FPHBr, respectively. Similarly, skin protein hydrolysates (SPHs) prepared using pepsin, papain, and bromelain individually were designated as SPHPe, SPHPa, and SPHBr, respectively. The protein contents of protein isolates and hydrolysates were determined using the modified Lowry method [24]. In the following bioactivity assays and rat study, the concentration (mg/mL) of protein hydrolysates applied reptresents the final protein concentration of samples.

### Degree of hydrolysis

The ortho-phthalaldehyde (OPA) method described by Charoenphun et al. [25] was used to estimate DH with some modifications. The freshly prepared reagent consisted of 6 mM OPA (dissolved in methanol) and 0.2% (v/v) 2-mercaptoethanol in 50 mM sodium tetraborate containing 1% (w/v) SDS. A 200-μL aliquot of the OPA reagent was added to 5 μL of standard (gly-gly-gly) or protein hydrolysate and mixed. The mixture was incubated for 100 s at room temperature and the absorbance was measured at 340 nm using multiplate reader (Multiskan Go, Thermo Fisher Scientific, Waltham, MA, USA). Acidic hydrolysis using 6 N HCl at 110°C for 24 h was used to determine the total amount of primary amino groups. DH was defined as the percentage of cleaved peptide bonds as follows:

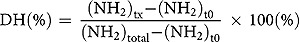

where (NH_2_)_tx_ is the number of free amino groups at X min and (NH_2_)_total_ is the total number of amino groups. (NH_2_)_t0_ represents the amount of free amino groups at 0 min of hydrolysis.

### Sodium dodecyl sulfate polyacrylamide gel electrophoresis (SDS-PAGE) analysis

Electrophoresis patterns of protein isolates and hydrolysates were analyzed using SDS-PAGE [26] with 4% stacking gel and 18% resolving gel. FPI, SPI, and their hydrolysates were dissolved in sample buffer (0.5 M Tris-HCl pH 6.8, glycerol, 10% SDS, w/v, 0.5% bromophenol blue, w/v, β-mercaptoethanol) at a concentration of 4 mg/mL and denatured at 95°C for 5 min. The loading volume was 10 μL in all sample lanes. After separation, the gel was stained with Coomassie Brilliant Blue R-250 for 30 min and then destained using water/methanol/acetic acid (7/2/1, v/v/v) followed by being scanned with E-Box VX5 (Vilber Lourmat, Paris, France). The standard protein marker (broad range molecular weight, Bio-Rad, Hercules, CA, USA) was used to construct a standard curve (10–250 kDa) for the MW estimation.

### Fractionation of pepsin-generated tilapia frame protein hydrolysate (FPHPe)

The FPHPe was further fractionated by ultrafiltration using an Amicon stirred ultrafiltration cell (Millipore Corporation, Bedford, MA, USA) with 1, 3, 5, and 10 kDa molecular weight cut-off (MWCO) membranes. Fractions with different MW cut-offs (< 1 kDa, 1–3 kDa, 3–5 kDa, and 5–10 kDa) were collected as previously described [27], lyophilized and stored at −20°C until use. The yields of hydrolysate fractions were calculated based on the dry weight of permeate against the dry weight of protein hydrolysate used for ultrafiltration.

### ACE inhibition assay

The effect of hydrolysates on inhibition of *in vitro* ACE activity was measured according to the method reported by Udenigwe et al. [28] with some modifications. A 0.5 mM N-[3-(2-furyl) acryloyl]-L-phenylalanyl glycyl glycine (FAPGG) was prepared in 50 mM Tris-HCl buffer to contain 0.3 M NaCl and adjusted to pH 7.5. The FAPGG was used as substrate and samples were dissolved in the same buffer as the FAPGG. When conducting assays, 170 μL of 0.5 mM FAPGG was mixed with 10 μL ACE (0.5 U/mL, final activity of 25 mU) and 20-μL sample. The rate of decrease in absorbance at 345 nm was recorded for 30 min at 37°C using Synergy H4 microplate reader (BioTek Instruments, Winooski, VT, USA). The buffer was used instead of sample solutions for the control (uninhibited reaction). ACE activity was expressed as the rate of reaction (ΔA/min) and inhibitory activity was calculated as:

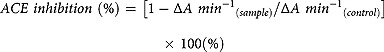

Where 
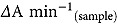
 and 
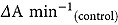
 represent ACE activity in the presence and absence of the peptides, respectively.

### Renin inhibition assay

*In vitro* inhibition of human recombinant renin was conducted using Renin Inhibitor Screening Assay Kit according to the method described by Girgih et al. [29]. Peptide fractions were dissolved in Tris-HCl buffer (50 mM, pH 8) containing 100 mM NaCl. The buffer was pre-warmed to 37°C prior to the reaction. Before the reaction, 1) 20 μL substrate, 151 μL assay buffer, and 19 μL Tris-HCl buffer were added to the background wells; 2) 20 μL substrate, 141 μL assay buffer, and 19 μL Tris-HCl buffer were added to the control wells; and 3) 20 μL substrate, 141 μL assay buffer, and 19 μL sample were added to the inhibitor wells. The reaction was initiated by adding 10 μL renin to the control and sample wells. The microplate was shaken for 10 s to mix and incubated at 37°C for 15 min, and the fluorescence intensity (FI) was then recorded at the excitation and emission wavelengths of 340 nm and 490 nm, respectively, on a fluorometric microplate reader (Spectra MAX Gemini, Molecular Devices, Sunnyvale, CA). The percentage of renin inhibition was calculated as follows:

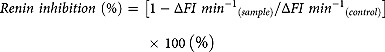

Where 
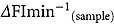
 and 
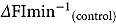
 represent renin activity in the presence and absence of the peptides, respectively.

### Evaluation of antihypertensive activity of tilapia frame peptides in SHR0073

Animal experiments were performed according to the protocol approved by the University of Manitoba Animal Protocols and Management Review Committee. Male spontaneously hypertensive rats (SHRs) were purchased at six weeks from Charles River (Montreal, PQ, Canada) and housed under a 12 h day and night cycle at 21°C and 50–55% humidity, with regular chow feed and tap water provided ad libitum. After one week acclimatization, SHRs were chronically implanted with Data Sciences International (DSI) HD-S10 telemetry transmitters (DSI, St. Paul, MN, USA), under anesthesia and analgesia, with all surgical procedures performed under sterile conditions. Rats were allowed two-week recovery period on ad libitum regular chow and water before oral gavage was performed. FPHPe-1 was dissolved in 1 mL of phosphate buffered saline (PBS) at a dose of 100 mg/kg body weight (BW) while captopril (an antihypertensive drug) was administered at a dose of 20 mg/kg BW, and the negative control rats received the saline solution only. Each group was orally gavaged with 1 mL of solution using a disposable plastic syringe. Real-time systolic and diastolic blood pressure (SBP and DBP) measurements, mean arterial pressure (MAP) and heart rates (HR) were collected in a quiet room with each rat cage placed on top of the receiver (Model RPC-1, DSI instruments, MN, USA) assigned to the implant. Data were recorded continuously at 10 min intervals for 24 h using Ponemah 6.1 data acquisition software (DSI instruments, MN, USA). An APR-1 atmospheric-pressure monitor (DSI instruments, MN, USA) was attached to the system to normalize the transmitted pressure values so that the recorded blood pressure signals were independent of atmospheric pressure changes. Results are reported as changes in values of the SBP, DBP, MAP and HR at 2, 4, 6, 8, 12 and 24 h minus their baseline measurements at time zero.

### Statistical analysis

All experiments were conducted in triplicate and analyzed by one-way analysis of variance (ANOVA) followed by Tukey’s multiple comparison test. The significance level of *p* < 0.05 was employed.

## Results and discussion

### Characterization of enzymatic hydrolysates

#### Degree of hydrolysis (DH)

**Figure 1.**
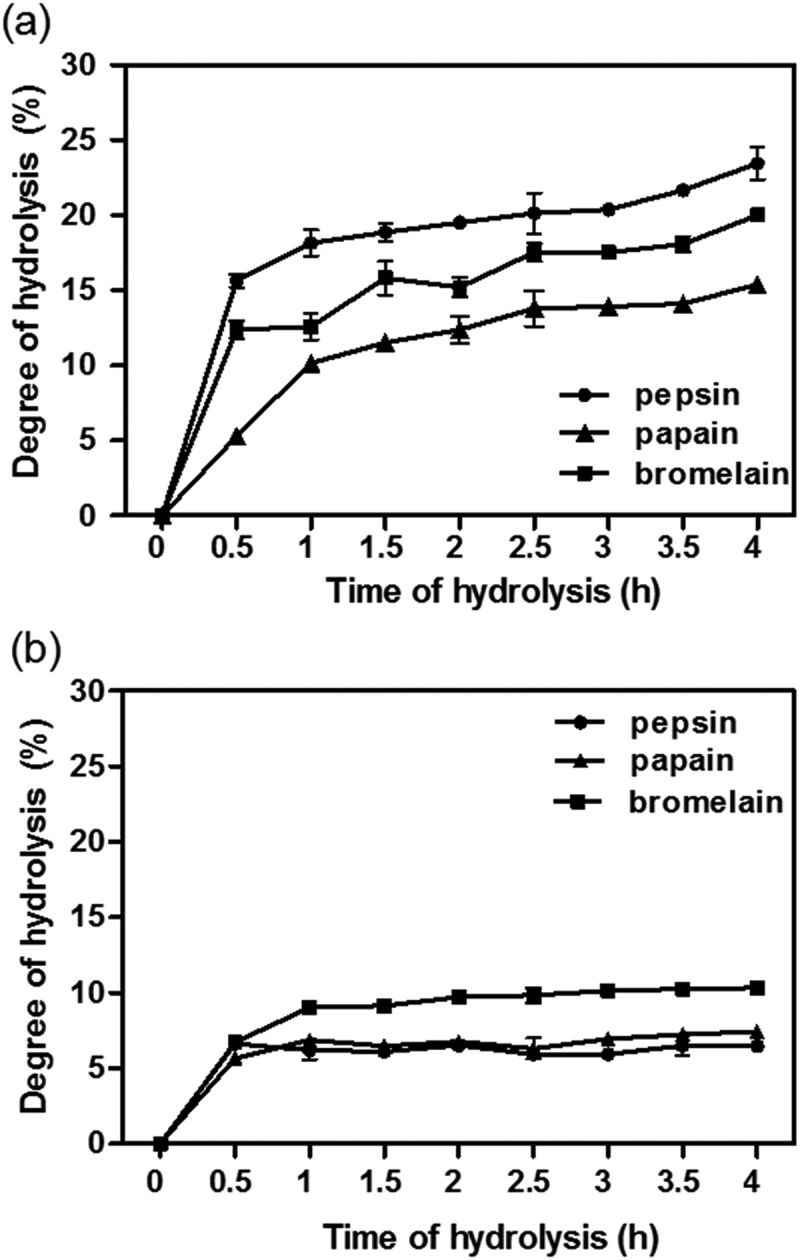
(a) Degree of hydrolysis (DH) of tilapia frame protein isolate (FPI) during enzymatic hydrolysis. (b) DH of tilapia skin protein isolate (SPI) during enzymatic hydrolysis.

During the enzymatic hydrolysis of FPI and SPI, the DH was measured at different time-points. During the first hour, the rate of pepsin and bromelain-catalysed FPI hydrolysis was higher than that of papain (Figure 1(a)). From 1–4 h, DH gradually increased at less rapid rates compared to the initial 30 min. For SPI hydrolysis, pepsin (SPHPe) and papain hydrolysates (SPHPa) had similar pattern and there was no noticeable increase in DH values after 30 min with a maximum of ~7%. In contrast, the DH of bromelain hydrolysate (SPHBr) was higher than that of SPHPe and SPHPa after after 30 min of hydrolysis but did not exceed ~10% (Figure 1(b)). Generally, the DH after 4 h was greater in frame hydrolysates (10–20%) than the skin hydrolysates (lower than 10%). The lower DH value for skin hydrolysates might be due to the triple-helical structure of collagen, which made SPI hard to cleave by the proteases except those belonging to the matrix metalloprotease (MMP) family like collagenases [30]. Collagen hydrolysates from other protein sources have been similarly characterized with low DH. For example, turkey head collagen hydrolysate produced using alcalase, flavourzyme or trypsin individually possessed fairly low DH values of 1–4% [31]. Even after subjecting the turkey collagen to dual-enzyme mixture or an enzyme cocktail comprising all the three proteases, the DH did not exceed 10%.

DH, yields and protein contents of tilapia enzymatic hydrolysates after 4 h hydrolysis are summarized in Table 1. The FPH produced by pepsin (FPHPe) and bromelain (FPHBr) possessed significantly higher (*p* < 0.05) DH, protein contents, and yields than papain-generated FPH (FPHPa). Moreover, the protein contents of FPHPe and FPHBr increased by about 10% compared to the FPI (PC: 75.35%), which suggests that the hydrolysate production led to reduced solubilization of non-protein FPI materials. In contrast, SPHPe and SPHPa with lower DH showed higher protein contents than the SPHBr but the hydrolysates had similar protein contents as the SPI (79.16%). There was no significant difference between the hydrolysate (SPHPe, SPHPa, and SPHBr) yields obtained from SPH digestion.

**Table 1.** Degree of hydrolysis (DH), protein content and yields of tilapia frame and skin protein hydrolysates after 4-h hydrolysis.

Samples	Enzyme (E/S^a^: 1%)	Degree of Hydrolysis (%)	Protein content (%)	Yield^b^ (%)
Frame protein hydrolysates (FPHs)	Pepsin (FPHPe)	23.46 ± 1.08^a^	84.93 ± 1.01^a^	80.66 ± 6.83^a^
	Papain (FPHPa)	15.38 ± 0.04^c^	68.88 ± 5.36^b^	56.42 ± 14.03^b^
	Bromelain (FPHBr)	20.01 ± 0.22^b^	89.05 ± 1.10^a^	67.85 ± 0.78^a^
Skin protein hydrolysates (SPHs)	Pepsin (SPHPe)	6.48 ± 0.10^e^	79.51 ± 8.37^a^	105.14 ± 14.09^a^
	Papain (SPHPa)	7.44 ± 0.20^e^	81.43 ± 3.33^a^	81.25 ± 17.47^a^
	Bromelain (SPHBr)	10.33 ± 0.15^d^	68.21 ± 2.80^b^	92.39 ± 0.40^a^

^a^E/S: the ratio of the weights of enzymes against substrates.

^b^The yield was calculated based on the dry weight of resultant hydrolysate against the dry weight of protein isolate used for hydrolysis.

Results are presented as mean ± standard deviation with triplicate measurements. For each column, values that contain different letters are significantly different at *p*< 0.05.

#### Protein/peptide patterns

**Figure 2.**
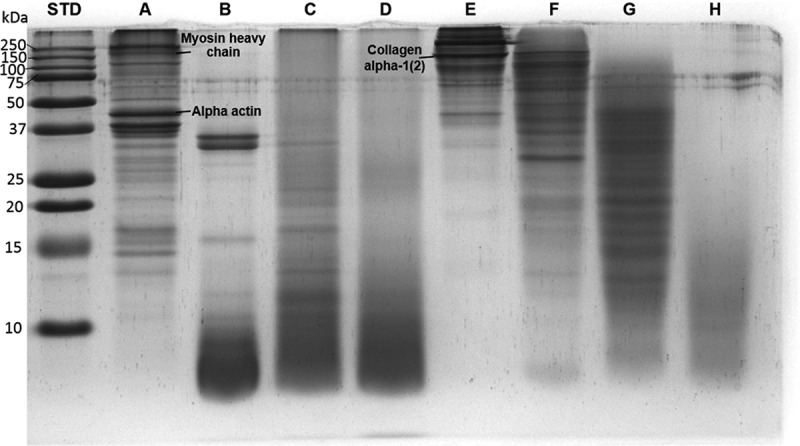
SDS-PAGE of samples from tilapia frame protein isolate (FPI, lane A), frame protein hydrolysates generated by pepsin (FPHPe, lane B), papain (FPHPa, lane C) and bromelain (FPHBr, lane D), skin protein isolate (SPI, lane E), and skin protein hydrolysates generated by pepsin (SPHPe, lane F), papain (SPHPa, lane G) and bromelain (SPHBr, lane H).

SDS-PAGE analysis was carried out to visualize the protein/peptide patterns of FPI, SPI, FPHs (FPHPe, FPHPa, and FPHBr), and SPHs (SPHPe, SPHPa, and SPHBr) (Figure 2). Multiple bands higher than 10 kDa were displayed in lane A (FPI) with much more intense bands from 37 to 250 kDa. According to the result of tilapia protein identification from FPI using proteomic strategy [2], the 37–50 kDa band in lane A may be related to α-actin (39.3 kDa) while myosin heavy chain (173.3 kDa) may appear in the 150–250 kDa band. Protein bands with MWs above 37 kDa were not observed in the lanes of FPHs (lane B, C and D), which suggests the degradation of identified proteins in FPI. Two obvious bands above 100 kDa in lane E may be related to different chain types of SPI collagen. The 100–150 kDa band could be collagen alpha-1(2) as we have previously identified [2].

Most peptides shown in lane B, C and D were lower than 10 kDa, which is consistent with extensive protein hydrolysis. In lane B, bands corresponding to MWs of myosin and actin disappeared, replaced by two distinct bands of MW around 25–37 kDa and a group of smaller peptides lower than 10 kDa. The polypeptide patterns of flaxseed protein hydrolysates reported by Karama´c et al. [32] also demonstrated that the enzymatic treatment resulted in the degradation of major proteins in flaxseeds. SPHPe (lane F) mainly consisted of peptides larger than 25 kDa while the MWs of SPHPa (lane G) ranged from 10 to 50 kDa. Protein band patterns of FPHs and SPHs were strongly related to the DH. FPHPe (lane B) with the highest DH seemed to contain the highest amount of low MW (< 10 kDa) peptides. A similar trend occurred in the protein hydrolysates of red tilapia (*Oreochromis niloticus*) fillet that as DH increased with time, peptide bands with MWs lower than 14.4 kDa became more obvious [23].

### Prediction of bioactive peptides derived from identified proteins by BIOPEP analysis

In the BIOPEP database, the frequency of bioactive fragments (A) in a protein chain equals the number of fragments with a given activity (a) within the protein divided by the number of amino acid residues (N) within this protein (A = a/N) [13]. The frequency of ACE-inhibitory peptides in SPI-derived collagen alpha-1(I) and collagen alpha-2(I) was 0.722 and 0.756, respectively. The frequency of peptides with the ACE-inhibitory activity was 0.350–0.414 in the proteins (myosin heavy chain, troponin T, creatine kinase, fructose-bisphosphate aldolase A and alpha actin) identified from FPI [2]. Accordingly, SPI might be a more excellent protein source of ACE-inhibitory peptides than FPI based on the in silico study.

Since encrypted peptides need to be released from precursor protein to possess activities, simulation of enzymatic digestion of FPI and SPI protein sequences using BIOPEP’s enzyme action tool was conducted. The numbers of ACE-inhibitory peptides liberated by pepsin, papain, bromelain, and a combination of digestive enzymes (pepsin, trypsin, and chymotrypsin A or C) are displayed in Table 2. In addition, the sequences as well as the number of each kind of ACE-inhibitory peptide potentially released are also summarized in the supplementary data (Table S1). The in silico results suggest that pepsin and papain released more ACE-inhibitory peptides from FPI than bromelain (Table 2(a)). On the other hand, in silico digestion of SPI by papain generated 129 and 121 ACE-inhibitory peptides, which were more than the numbers released by pepsin (54 and 51) and bromelain (97 and 85) (Table 2(b)). Although computer aided simulation is a useful research tool for choosing the most appropriate enzymes, actual *in vitro* or *in vivo* experiments are still necessary because external factors may be involved during protein hydrolysis, which limits utility of the in silico methods.

**Table 2.** Number of predictive angiotensin converting enzyme (ACE)-inhibitory peptides released from identified tilapia (a) frame proteins and (b) skin proteins using BIOPEP’s enzyme action tool.

(a)	Alpha actin*	Myosin heavy chain^a,^*	Creatine kinase^b,^*	Troponin T*	Fructose-bisphosphate aldolase A*
Pepsin	13	68	16	21	11
Papain	18	61	18	15	13
Bromelain	12	38	5	5	10
Pepsin+Trypsin+ Chymotrypsin A	18	76	24	18	10
Pepsin+Trypsin+ Chymotrypsin C	22	78	27	16	11

(b)	Collagen alpha-1(I) chain^c,^ *	Collagen alpha-2(I) chain^d,^ *
Pepsin	54	51
Papain	129	121
Bromelain	97	85
Pepsin+Trypsin+ Chymotrypsin A	104	96
Pepsin+Trypsin+ Chymotrypsin C	221	214

^a^Myosin heavy chain, fast skeletal muscle-like. ^b^Creatine kinase M-type-like. ^c^Collagen alpha-1(I) chain-like isoformX2. ^d^Collagen alpha-2(I) chain-like isoformX1. *Protein sequences of the tilapia skin proteins shown in this table were all identified in our previous study [2].

### In vitro ACE inhibitory activity of FPH and SPH

**Figure 3.**
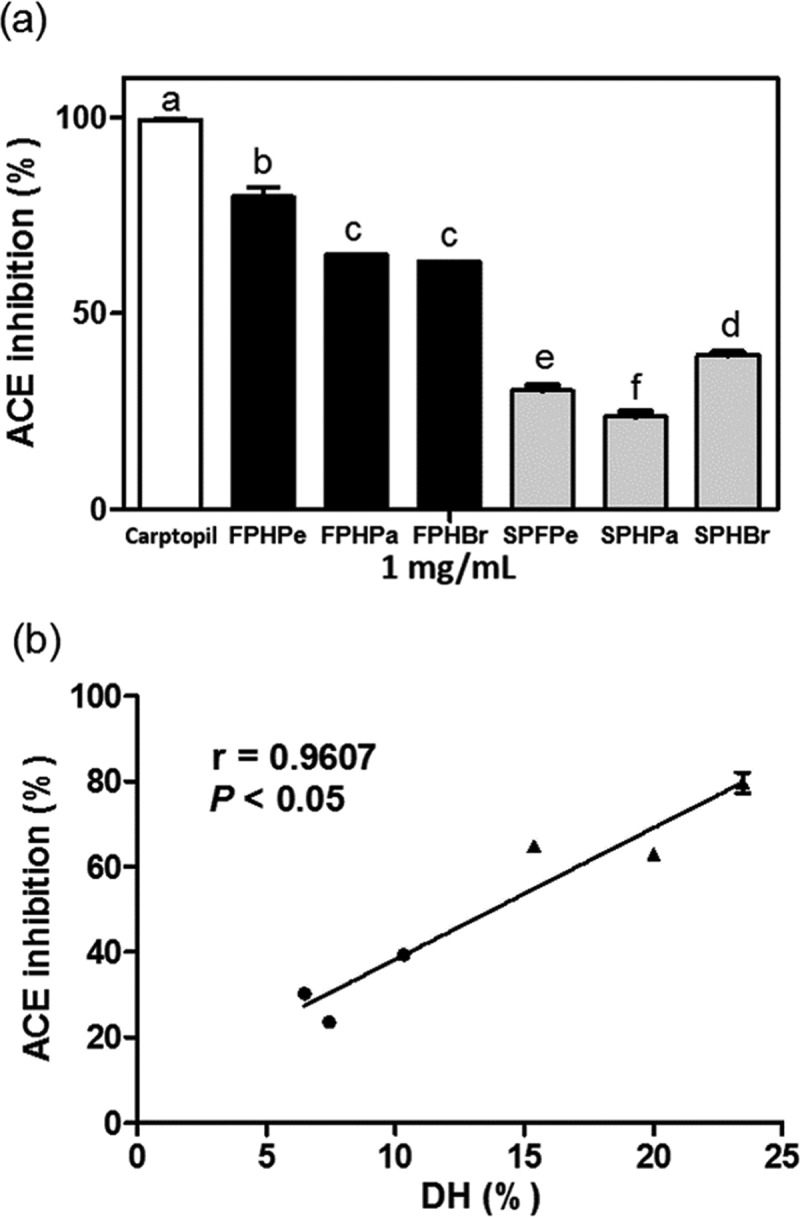
(a) *In vitro* Angiotensin-I converting enzyme (ACE) inhibitory activities of frame protein hydrolysates (FPHs: FPHPe, FPHPa, and FPHBr) and skin protein hydrolysates (SPHs: SPHPe, SPHPa, and SPHBr) generated by pepsin, papain, or bromelain. Bars with different letters are significantly different (*p **< ***0.05). (b) The correlation plot between degree of hydrolysis (DH) and ACE inhibitory activities for FPHs (●) and SPHs (▲). The Pearson’s r value is 0.9607 and *p **< ***0.05 represents the correlation is significant.

The ACE-inhibitory activity of hydrolysates was evaluated and results showed that all FPHs exhibited significantly stronger activity (*p* < 0.05) than SPHs (Figure 3(a)). Captopril almost completely inhibited ACE activity (99.3%) while FPHPe with the highest DH revealed 79.7% ACE-inhibitory activity at the same 1 mg/mL concentration. Correspondingly, there was a significant linear relationship between the DH and the ACE inhibitory activity (Figure 3(b)). Based on the in silico result in Table 2(b), FPHPa seemed to have greater activity than FPHBr because of more ACE-inhibitory peptides released. However, there was no significant difference (*p* > 0.05) between the *in vitro* inhibitory properties of these two hydrolysates. This might be due to the relatively lower DH of FPHPa causing a restricted liberation of the ACE-inhibitory peptides. Similarly, SPHBr with higher DH than SPHPe and SPHPa also showed stronger ACE-inhibitory activity (39%). Previous research indicated Cryotin-F and Flavourzyme hydrolysates of tilapia fillet with the 25% DH possessed greater ACE-inhibitory activity than those with the DH value of 7.5% [7]. Thus, it was likely that ACE-inhibitory activity observed for FPHs and SPHs was determined mostly by their peptide size.

The IC_50_ value of FPHPe was 0.57 mg/mL, which was similar to the IC_50_ value of pepsin-catalyzed krill (*Euphausia superba*) protein hydrolysate (0.58 mg/mL) [33] but lower than that of <5 kDa chymotrypsin hydrolysate from yellowfin sole (*Limanda aspera*) frame (0.88 mg/mL) [34]; the ACE-inhibitory activity of FPHPe was also stronger than the pepsin hydrolysate from sea squirt (*Styela plicata*) (IC_50_: 2.43 mg/mL) [35]. On the other hand, while SPHs showed weaker ACE inhibition effects, hydrolysis by serial protease-treatments may be a feasible way to overcome this obstacle. This was illustrated by the following sequential hydrolysis of Alaska Pollack skin gelatin extracts using alcalase, pronase E, and collagenase in a three-step recycling membrane reactor, which produced ACE-inhibitory hydrolysate with a low IC_50_ value of 0.63 mg/mL [36]. The 0.63 mg/mL value is twice as potent as the single enzyme hydrolysate (IC_50_: 1.4 mg/mL).

### ACE and renin inhibitory activities of peptide fractions from FPHPe

The ultrafiltration system using 1–10 kDa MWCO membranes is usually applied to separate bioactive peptides into fractions with different molecular weights [37,38]. This is because the size of ACE-inhibitory peptides is generally between 2 and 30 amino acids [39]. FPHPe was selected for further purification using an ultrafiltration system because it exhibited superior ACE-inhibitory activity among the FPHs and SPHs. According to the outcome of BIOPEP analysis, more than 70% of the ACE-inhibitory peptides from pepsin-hydrolyzed frame proteins were dipeptides and ~18% were tripeptides. The protein contents of <1 kDa, 1–3 kDa, and 3–5 kDa hydrolysate fractions were 91.47%, 91.39%, and 97.19%, respectively, which were all higher than FPI and FPHPe. Both protein content (86.42%) and yield (2.78%) of 5–10 kDa hydrolysate fraction were the lowest compared to other fractions. The yields of <1 kDa, 1–3 kDa, and 3–5 kDa hydrolysate fractions were 7.63%, 14.85%, and 7.42%, respectively.

**Figure 4.**
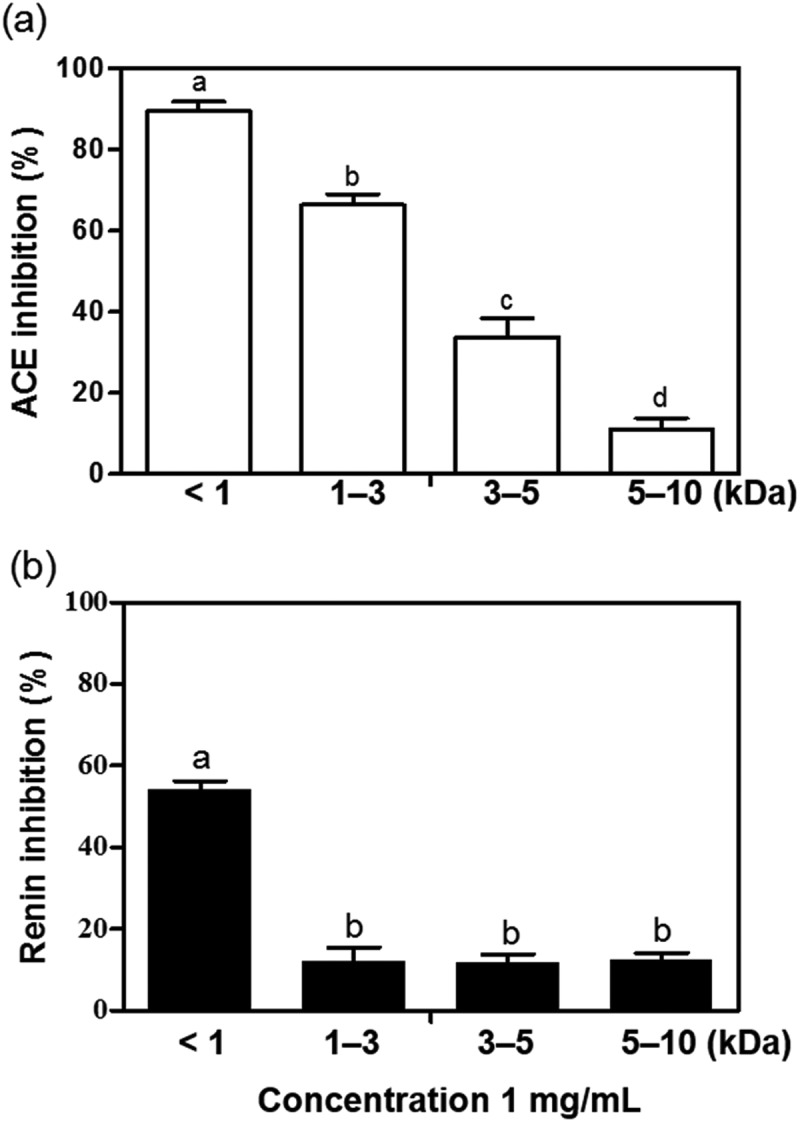
(a) *In vitro* angiotensin-I converting enzyme (ACE) inhibitory activities of the ultrafiltration fractions of pepsin-hydrolyzed frame protein hydrolysate (FPHPe) at of 0.6 mg/mL. (b) *In vitro* renin inhibitory activities of the fractions from FPHPe at of 1 mg/mL. Bars with different letters have significantly (*p **< ***0.05) different mean values.

Results of the ACE inhibition assay revealed that the <1 kDa ultrafiltration fraction from FPHPe (FPHPe1) showed the strongest inhibitory effect on ACE activity while the potency decreased as the MWs of peptides increased (Figure 4(a)). The IC_50_ values of <1 kDa, 1–3 kDa, 3–5 kDa, and 5–10 kDa fractions were 0.41 mg/mL, 0.55 mg/mL, 0.79 mg/mL, and 0.83 mg/mL, respectively. The IC_50_ value of FPHPe1 was similar to 0.46 mg/mL reported for the Alaska pollack frame <1 kDa protein hydrolysate [40], but higher than the 0.19 mg/mL for krill [33]. With respect to renin inhibitory activity, FPHPe1 had considerably higher (*p* < 0.05) inhibitory effect (54.28%) than the larger peptide sizes at 1 mg/mL concentration (Figure 4(b)). The hydrolysates from kidney bean protein [41] and bovine hemoglobin [42] displayed a similar trend. Since FPHPe1 showed a better capacity to inhibit both ACE and renin activities than the other fractions, the hypotensive effect of this peptide fraction in SHR was subsequently investigated.

### Antihypertensive effect of FPHPe1 in SHR

The peptides must be intact and absorbed through the intestine and reach the target enzymes in an active form to produce antihypertensive effects *in vivo*. Short peptides consisting of two or three amino acids are absorbed more quickly than free amino acids [43]. Although larger peptides (10–15 amino acids) can also be absorbed through the intestine to generate biological effects, the potency of the peptide decreases as the chain length increases [44]. After *in vivo* hydrolysis, the numbers and sequences of bioactive peptides may change due to further cleavage of digestive enzymes, resulting in the enhancement or loss of activities. The numbers of pepsin-generated ACE-inhibitory peptides from alpha-actin, myosin heavy chain, and creatine kinase increased when the in silico digestion was conducted along with trypsin and chymotrypsin (Table 2(a)), which simulated gastrointestinal tract digestion. Additionally, under the simultaneous actions of trypsin and chymotrypsin the renin inhibitor KF (EC_50_: 17.84 μM) from pepsin-digested myosin and troponin T was supplemented by the other renin inhibitory peptide IR, which possesses lower IC_50_ value (9.2 μM).

**Figure 5.**
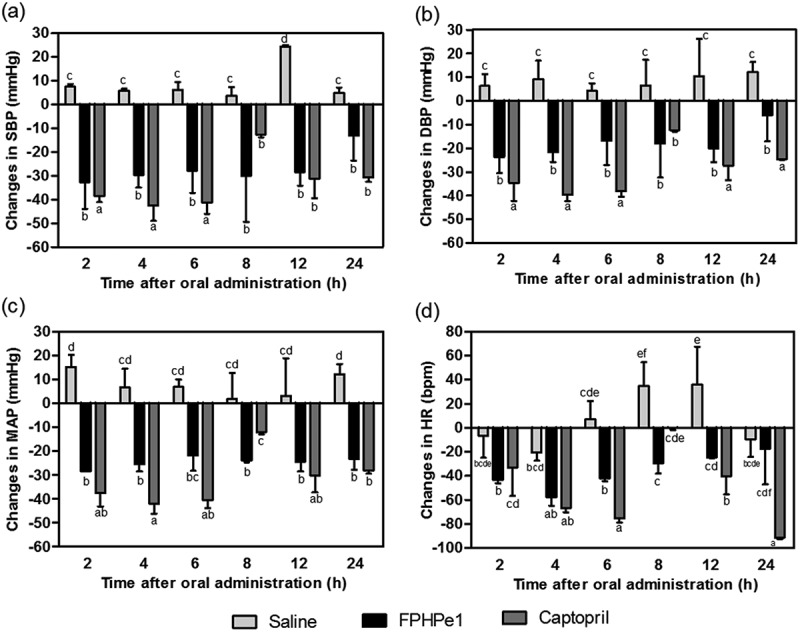
The effect of the < 1 kDa ultrafiltration fraction from pepsin-hydrolyzed frame protein hydrolysate (FPHPe1) on (a) systolic blood pressure (SBP), (b) diastolic blood pressure (DBP), (c) mean arterial pressure (MAP), and (d) heart rate (HR) of spontaneously hypertensive rats (SHRs) after oral gavage. Different letters above the bars indicate significant differences (*p **< ***0.05).

The antihypertensive effect of FPHPe1 was evaluated based on the changes in physiological parameters of hypertension including SBP, DBP, MAP and HR after oral administration to SHRs. Figure 5 shows that the saline solution was ineffective reducing SBP, DBP, and MAP of SHRs during the 24 h after administration, whereas the FPHPe1 showed fast-acting effects in SHRs. The maximal reduction of SBP, DBP, and MAP caused by FPHPe1 appeared after 2 h, which was – 33, – 24, and – 28 mm Hg, respectively. The decrease of HR also happened with a maximum reduction of 58 beats per min after 4 h. On account of no significant difference (*p* > 0.05) between the changes in SBP, DBP, and MAP at different timepoints after oral gavage with peptides, FPHPe1 was considered to provide a rapid and persistent antihypertensive effect in SHRs. Furthermore, the *in vivo* antihypertensive property of FPHPe1 at 100 mg/kg BW was quite identical to the hypotensive effect in SHRs treated with captopril (20 mg/kg BW) as changes of SBP, MAP and HR in SHRs did not show a significant difference (*p* > 0.05). The positive result of the rat study has established that the active FPHPe1 peptides may have survived gastrointestinal digestion or were hydrolyzed to give other active sequences, all of which contributed to the excellent blood pressure-lowering effects in SHRs.

## Conclusions

Protein hydrolysates prepared from tilapia coproducts including frame and skin demonstrated varying degrees of *in vitro* inhibitory activities against renin and ACE. FPHs with higher DH showed stronger *in vitro* ACE-inhibitory effect than SPHs even though in silico proteolysis suggested a higher number of ACE-inhibitory peptides in the latter. Fraction FPHPe1 (<1 kDa) exhibited greater ACE and renin inhibitory activities, which is consistent with the fact that most bioactive peptide sequences identified within tilapia protein sequences in the BIOPEP database are less than 1 kDa. The rapid reduction in blood pressure and the long duration of the antihypertensive effect exhibited by FPHPe1 in SHRs indicate that the protein hydrolysate from tilapia frame was able to develop into a promising ingredient for the formulation of antihypertensive functional foods or nutraceuticals. However, various factors such as the DH, diverse IC_50_ values, and the unavailability of peptide information in databases could have contributed to the observed inconsistency between experimental and theoretical (in silico) results. This study has demonstrated that proteomics strategies for protein identification coupled with BIOPEP analysis of potential bioactive peptides is a feasible way to select appropriate protein sources or enzymes when producing bioactive peptides.

## Supplementary Material

F_N_research_2017_Supplementary_Table.docx
