# Supplementary material for: Antihypertensive properties of tilapia (Oreochromis spp.) frame and skin enzymatic protein hydrolysates
Source: Food Nutr Res. 2017 Oct 24;61(1):1391666. doi: 10.1080/16546628.2017.1391666 (PMC5678373; doi:10.1080/16546628.2017.1391666)
Supplement: F_N_research_2017_Supplementary_Table.docx [file ZFNR_A_1391666_SM3015.docx]

Antihypertensive properties of tilapia (*Oreochromis spp.*) frame and skin enzymatic protein hydrolysates

Hsin-Chieh Lin^a^, Adeola M. Alashi^b^, Rotimi E. Aluko^b^, Bonnie Sun Pan^a^ and Yu-Wei Chang^a^*

^a^Department of Food Science, National Taiwan Ocean University, Keelung, Taiwan; ^b^Department of Human Nutritional Sciences, University of Manitoba, Winnipeg, MB, Canada

*Corresponding author: Yu-Wei Chang

*Tel: +886-2-2462-2192 ext. 5152*

*Fax: +886-2-2463-4203*

*E-mail: [bweichang@mail.ntou.edu.tw](mailto:bweichang@mail.ntou.edu.tw)*

***Supplementary Table S1***

Table S1. The angiotensin converting enzyme (ACE)-inhibitory peptides potentially released from (a) identified frame proteins (alpha actin, myosin heavy chain, creatine kinase M-type-like, troponin T, and fructose-bisphosphate aldolase A) and (b) identified skin proteins (collagen alpha-1(I) chain-like isoformX2 and collagen alpha-2(I) chain-like isoformX1) by proteases using BIOPEP analysis are summarised.

| **(a)** | | **Alpha actin** | | | | |  |  |
| --- | --- | --- | --- | --- | --- | --- | --- | --- |
|  | | No. of  peptides | | Peptide sequence | | |  |  |
| Pepsin | | 13 | | VF(1), MY(1), RF(1), GY(1), GPL(1), GF(1), DA(1), NF(1), SF(1), KY(1), TE(2), KE(1) | | |  |  |
| Papain | | 18 | | MY(1), PR(2), IVR(1), IWH(1), IA(1), LA(1), FR(1), VG(1), MG(1), QG(1), SG(2), PG(2), QK(1), SY(1), ME(1) | | |  |  |
| Bromelain | | 12 | | MY(1), IA(1), LA(1), VG(1), MG(1), SG(2), EG(1), PG(1), QK(1), DG(1), SY(1) | | |  |  |
| Pepsin+Trypsin+  Chymotrypsin A | | 18 | | VF(1), MY(1), GY(2), PR(2), IVR(1), GPL(1), IW(1), VK(1), GF(1), GR(1), DA(1), NF(1), SY(1), SF(1), TE(2) | | |  |  |
| Pepsin+Trypsin+  Chymotrypsin C | | 22 | | VF(1), VY(1), GY(2), IVR(1), GP(1), IW(1), VK(1), GF(1), GR(1), DA(2), GQ(1), NF(1), SY(1), SF(2), IE(1), TE(2), TQ(1), HP(1) | | |  |  |
|  | | **Myosin heavy chain, fast skeletal muscle-like** | | | | |  |  |
|  | | | | Peptide sequence | | |  |  |
| Pepsin | | 68 | | RL(5), GPA(1), RY(1), IY(1), KW(1), RF(1), IA(1), RA(8), GF(2), IF(1), GA(4), HL(3), DA(2), GQ(2), GE(3), KY(1), KF(1), KL(6), KA(5), CF(1), IE(5), VE(1), TE(3), TQ(1), ME(3), KE(5) | | |  |  |
| Papain | | 61 | | IR(1), IY(2), PR(1), VK(8), IA(1), LA(4), VG(2), FG(3), DA(2), SG(3), LG(2), NG(2), VR(3), QK(6), SY(1), NK(2), MDLA(1), IE(7), VE(2), TE(3), ME(4), PH(1) | | |  |  |
| Bromelain | | 38 | | IY(2), VK(3), IA(1), LA(4), RA(1), VG(1), FG(1), DA(2), SG(2), EG(1), EA(4), QK(6), NK(2), MDLA(1), EK(2), HK(2), SVY(1), FDK(1), LEK(1) | | |  |  |
| Pepsin+Trypsin+  **Table S1(a) (Continued)**  Chymotrypsin A | | 76 | | IR(5), GPA(1), IY(1), IY(1), MF(1), PR(2), VK(8), IA(2), GF(3), IF(1), GA(4), HL(3), GR(1), DA(3), GQ(4), GK(8), GE(3), VR(3), NF(1), SY(1), NK(2), CF(1), IE(9), VE(2), TE(4), TQ(1), ME(5), SVY(1) | | |  |  |
| Pepsin+Trypsin+  Chymotrypsin C | | 78 | | IR(1), IY(2), VY(1), GP(1), VK(8), IA(3), ITTNP(1), IP(1), GF(3), IF(1), GM(2), GA(4), GL(2), HL(3), GR(1), DA(3), GQ(4), GK(8), GE(3), VR(3), NF(1), SY(1), NK(3), CF(1), IE(9), VE(3), TE(4), TQ(1) | | |  |  |
|  |  |  | | **Creatine kinase M-type-like** | | |  |  |
|  | |  | | Peptide sequence | | |  |  |
| Pepsin | | 16 | | RL(2), VF(2), VW(1), VY(1), GY(1), PL(1), IA(1), IF(1), HL(1), NY(1), SY(1), KL(1) , VE(1), ME(1) | | |  |  |
| Papain | | 18 | | VY(1), VFK(1), IWH(1), VK(2), LG(2), TG(3), VR(1), QK(1), SY(1), NK(1), IE(1), VE(2), ME(1) | | |  |  |
| Bromelain | | 5 | | VK(1), TG(2), QK(1), HK(1) | | |  |  |
| Pepsin+Trypsin+  Chymotrypsin A | | 24 | | VF(2), VW(1), VY(1), GY(1), PL(1), IA(1), GF(1), IF(1), HL(1), GK(2), GE(2), VR(1), NY(1), SY(1), NK(1), IE(1), VE(2), ME(1), HK(1) , TF(1) | | |  |  |
| Pepsin+Trypsin+  Chymotrypsin C | | 27 | | VF(2), VW(1), VY(1), GY(1), VSP(1), IA(1), IP(1), GF(1), IF(1), GM(1), HL(1), GK(2), GE(2), VR(1), NY(2), SY(1), NK(1), IE(1), VE(3) , HK(1), TF(1) | | |  |  |
|  | |  | | **Troponin T** | | |  |  |
|  | |  | | Peptide sequence | | |  |  |
| Pepsin | | 21 | | RL(1), IA(1), RA(2), DA(3), GE(1), KY(1), KF(1), KL(1), VE(2), TE(2), PQ(1), ME(1) , KE(4) | | |  |  |
| Papain | | 15 | | IR(1), VG(1), DA(3), VR(1), QK(1), IE(2), VE(3), TE(3) | | |  |  |
| Bromelain | | 5 | | DA(1), EK(4) | | |  |  |
| Pepsin+Trypsin+  Chymotrypsin A | | 18 | | IR(1), PL(1), IA(1), GA(1), DA(3), GE(1), VR(1), NK(1), IE(2), VE(2), TE(2), PQ(1), ME(1) | | |  |  |
| Pepsin+Trypsin+  Chymotrypsin C | | 16 | | IR(1), IA(1), IP(1), GA(1), DA(3), GE(1), VR(1), NK(1), IE(2), VE(2), TE(2) | | |  |  |
|  | |  | | **Fructose-bisphosphate aldolase A** | | |  |  |
| **Table S1(a) (Continued)** | |  | | Peptide sequence | | |  |  |
| Pepsin | | 11 | | RY(1), KW(1), VY(1), IA(1), GL(1), SY(1), KA(2), PQ(1), KE(1), TF(1) | | |  |  |
| Papain | | 13 | | LY(1), VY(2), IA(1), LA(2), WG(1), NG(1), PG(1), QK(2), DG(1) | | |  |  |
| Bromelain | | 10 | | RY(1), LY(1), VY(1), LA(1), RA(2), WG(1), PG(1), QK(1), DG(1) | | |  |  |
| Pepsin+Trypsin+  Chymotrypsin A | | 10 | | VY(1), VK(1), IA(1), GL(1), GR(1), SY(1), PQ(1), HK(1), TF(1), DGL(1) | | |  |  |
| Pepsin+Trypsin+  Chymotrypsin C | | 11 | | VY(1), VK(1), IA(1), VP(1), GL(1), GR(1), GK(1), SY(1), HK(1), TF(1), DGL(1) | | |  |  |
| **(b)** | | **Collagen alpha-1(I) chain-like isoformX2** | | | | |  |  |
|  | | No. of  peptides | | Peptide sequence | | |  |  |
| Pepsin | | 54 | | GPA(9), MF(1), IRA(1), PGL(2), PL(1), RA(2), GF(2), GA(22), GL(3), DA(1), GQ(1), GE(5), SF(2) , KA(1), TQ(1) | | |  |  |
| Papain | | 129 | | IR(2), MY(1), PR(8), PLG(2), LPG(9), VK(1), IA(3), LA(2), VG(5), DA(1), MG(1), QG(7), SG(10), LG(3), TG(10), NG(4), PG(50), VR(1), DG(8), VE(1) | | |  |  |
| Bromelain | | 97 | | MY(1), HY(1), PLG(2), LPG(9), VK(1), IA(3), LA(1), RA(1), VG(4), DA(1), QG(5), SG(7), LG(3), TG(4), EG(1), EA(7), NG(2), PG(35), DG(5), PG(35), DG(5), EK(3), HK(1) | | |  |  |
| Pepsin+Trypsin+  Chymotrypsin A | | 104 | | IR(2), GPA(13), MF(1), PGL(2), PL(1), VK(1), GF(8), GA(33), GL(5), GR(5), DA(1), GQ(3), GK(3), GE(21), SF(2), VE(1), TQ(1), TF(1) | | |  |  |
| Pepsin+Trypsin+  Chymotrypsin C | | 221 | | IR(2), GY(1), GP(78), VK(1), VP(1), GF(12), GM(2), GA(52), GL(11), GR(7), DA(1), GQ(5), GK(8), GE(34), SF(2), VE(1), TQ(1), TF(2) | | |  |  |
|  | |  | | | **Collagen alpha-2(I) chain-like isoformX1** | | |  |
|  | |  | | Peptide sequence | | | |  |
| Pepsin | | 51 | | GPA(10), GPL(1), PL(1), RA(3), GF(1), GA(17), GL(5), GQ(1), GE(8), SF(1), IE(1), TQ(5), TF(1) SF(2) , KA(1), TQ(1) | | | |  |
| Papain | | 121 | | PR(14), FY(1), PLG(2), LPG(8), VK(2), LA(8), VG(7), IG(1), FG(1), DA(1), QG(2), SG(7), LG(2), TG(11), NG(2), PG(39), VR(1), DG(9), NK(1), TE(1), PH(1) | | | |  |
| Bromelain | | 85 | | FY(1), PLG(2), LPG(8), VK(2), LA(8), RA(2), VG(4), DA(1), HG(3), SG(7), LG(1), TG(7), EG(4), EA(3), NG(2), PG(19), DG(6), EK(4), HK(1) | | | |  |
| Pepsin+Trypsin+  Chymotrypsin A | | 96 | | GPA(12), VF(1), GY(1), PGL(1), PL(1), GF(2), GA(27), GL(11), GR(6), GQ(1), GK(2), GE(26), SF(1), NK(1), IE(1), TQ(1), TF(1) | | | |  |
| Pepsin+Trypsin+  Chymotrypsin C | | 214 | | VF(1), GY(1), GP(81), GF(6), GM(5), GA(45), GL(19), GR(9), GQ(2), GK(6), GE(32), SHP(1), SF(1), NK(1), IE(1), TQ(1), HP(1), TF(1) | | | |  |

**Table S1(b) (Continued)**

**Table S1 (b) (Continued)**
